# Pharmaceutical Interventions for the Management of Hemorrhagic Shock in Hepatic Surgery: An Experimental Swine Model Outcome

**DOI:** 10.7759/cureus.69734

**Published:** 2024-09-19

**Authors:** Ilianna I Kougia, Panteleimon Vassiliu, Dina G Tiniakos, Tzortzis Nomikos, Apostolos E Papalois, Vassilios Smyrniotis, Nikolaos Arkadopoulos

**Affiliations:** 1 Breast Surgery, Leto Maternity Hospital, Athens, GRC; 2 Surgery, 4th Surgical Clinic, Attikon University Hospital, National and Kapodistrian University of Athens, Athens, GRC; 3 Pathology, Aretaieio Hospital, National and Kapodistrian University of Athens, Athens, GRC; 4 Nutrition and Dietetics, Harokopio University, Athens, GRC; 5 Education and Training, Healthcare Education and Advanced Learning (HEAL Academy) by Hellenic Healthcare Group (HHG), Athens, GRC

**Keywords:** antioxidant, bucillamine, hemorrhagic shock, hepatic surgery, ischemia reperfusion, oxidative stress, resuscitation, swine model, trauma, valproic acid

## Abstract

Introduction

During abdominal trauma or major hepatic surgery, the liver can be subjected to hypoxic conditions due to hemorrhage, leading to various degrees of ischemic injury to hepatocytes. Hemorrhagic shock, a critical and life-threatening condition, often complicates hepatic surgery due to massive blood loss, resulting in inadequate tissue perfusion and oxygenation. The challenge in managing hemorrhagic shock in hepatic surgery is heightened by the liver's unique blood supply and its crucial role in coagulation. Effective treatment requires a multifaceted approach, including surgical intervention, blood transfusions, and pharmaceutical therapy to stabilize hemodynamics and promote coagulation. Additionally, reperfusion of the liver after resuscitation can cause severe injury through inflammatory and oxidative pathways, potentially leading to multiple organ dysfunction. This study examines the effectiveness of combining standard fluid therapy with antioxidants (bucillamine and valproic acid) in mitigating oxidative stress and reducing hepatocyte damage in the context of hemorrhagic shock.

Method

Thirty male swine were randomly divided into four groups as follows: Group A (n=6/control group), Group B (n=8), Group C (n=8), and Group D (n=8). A resection of the left liver lobe was performed, followed by the controlled loss of a specific volume of blood until the mean arterial pressure dropped to between 30 and 40 mmHg. The animals were maintained in this state of hypovolemic shock for 50 minutes, followed by IV administration of fluids. Additionally, pharmaceutical agents were administered to Groups B (bucillamine), C (valproic acid), and D (combination of bucillamine and valproic acid). The experiment lasted six hours in total.

Results

The study showed that animals treated with pharmaceutical agents like bucillamine and valproic acid and their combination demonstrated significantly reduced serum levels of protein carbonyls (PCs) and thiobarbituric acid reactive substances (TBARS) compared to the control group. Furthermore, histological evaluation of liver tissues revealed that treated animals showed reduced histopathological signs of injury, suggesting that the pharmaceutical agents not only lowered biochemical markers of oxidative stress but also provided a protective effect against liver damage in the context of hemorrhagic shock.

Conclusion

These findings suggest that incorporating antioxidant therapy into resuscitation protocols may offer substantial benefits in mitigating hepatic damage associated with hemorrhagic shock. This approach could potentially improve outcomes in clinical settings where oxidative stress plays a critical role in injury progression by better understanding the pathophysiology of this complex and dynamic process.

## Introduction

Hemorrhagic shock (HS) is characterized by severe hypovolemia resulting from extensive blood loss. This leads to decreased cardiac output and inadequate tissue perfusion. The body's compensatory mechanisms, such as vasoconstriction and tachycardia, initially attempt to maintain perfusion to vital organs. However, prolonged hypoperfusion results in cellular hypoxia, metabolic acidosis, and ultimately organ failure if not promptly addressed [1].

In the context of hepatic surgery, the risk of HS is heightened due to the liver's extensive vascular network. The liver receives about 25% of the cardiac output, with blood supplied by the hepatic artery and portal vein. Surgical manipulation or injury to these vessels can result in significant blood loss. Additionally, the liver plays a crucial role in synthesizing clotting factors, and hepatic dysfunction can exacerbate coagulopathy, complicating hemorrhage control [2].

The significant blood loss that often occurs during liver surgery, especially in major hepatectomies and transplants, is a major cause of perioperative mortality and morbidity [3]. Similarly, HS due to liver trauma remains the leading cause of death in patients with severe abdominal organ injuries [4].

Tissue hypoxia due to hypoperfusion during HS and the damage caused by reperfusion lead cells to a state of oxidative stress, which directly impacts their function and life cycle, inducing mechanisms of apoptosis and necrosis [5]. Reactive oxygen species (ROS) generated during oxidative stress can lead to lipid peroxidation, mitochondrial dysfunction, and the activation of pro-inflammatory pathways, exacerbating liver injury.

In addition to ischemia-reperfusion injury (IRI), it has also been shown that the large volume of fluids (crystalloids, colloids, and blood products) required to resuscitate HS is an adverse prognostic factor [6]. Specifically, the administration of large volumes of crystalloid solutions can lead to coagulation disorders, thereby worsening bleeding [7], acute renal failure, adult respiratory distress syndrome (ARDS), and secondary abdominal compartment syndrome, conditions that increase morbidity and therefore affect the outcome of patients.

Thus, in the last decade, the practice of ‘hypotensive resuscitation’ has become increasingly accepted, and new strategies are being designed to minimize the need for intravascular volume restoration [8,9].

Various pharmaceutical agents have been studied that, by intervening in oxidation and cellular apoptosis mechanisms, can minimize the damage from oxidative stress [10]. Compounds such as N-acetylcysteine, vitamin E, and superoxide dismutase mimetics have shown potential in mitigating oxidative stress and reducing liver injury in experimental models.

In our experiment, the administration of two substances, the antioxidant agent bucillamine (BA) and the histone deacetylase inhibitor valproic acid (VPA), as well as their combination, was studied. They were administered during the resuscitation of HS after partial hepatectomy in a swine model, with the goal being to evaluate whether the application of such a resuscitation protocol can be proven beneficial when it comes to HS due to hepatic trauma or surgery.

## Materials and methods

The study was completed at the 4th Surgical Clinic, Attikon University Hospital, National and Kapodistrian University of Athens, Athens, Greece, and the experiments were conducted at the Experimental, Educational, and Research Center ELPEN.

Experimental procedure

Animals

The protocol of the experiment was approved by the Hellenic Veterinary Services (license No. 954/02-03-2018). Male swine aged one year and weighing 30 ± 4 kg were used for the experimental study.

Animals that exhibited hypotension (BP < 80/40 mmHg, MAP < 60 mmHg) and leukocytosis (Leu > 20,000 cells/μL) during the clinical-laboratory examination before the start of the experimental procedure (blood sampling during carotid catheterization) were excluded from the study.

Living conditions: The animals were provided by the same breeder (Validakis, Koropi, Greece) and brought to the research facilities of the Experimental, Educational, and Research Center ELPEN (European Reference Number EL 09 BIO 03) one week prior to the experiment. They were housed in steel cages within a temperature-controlled environment and had access to food and water. Twelve hours before the experiment, they were fasted, having access to water only. The study followed the Animal Research: Reporting of In Vivo Experiments (ARRIVE) guidelines, ensuring the replacement, refinement, and reduction of animals used in research.

Sample size: Thirty laboratory animals were randomly (stratified randomization) divided into four groups, according to the research protocol, as follows:

Group A (n=6): HS and resuscitation with fluid administration only. Group B (n=8): HS and resuscitation with fluids and BA. Group C (n=8): HS and resuscitation with fluids and VPA. Group D (n=8): HS and resuscitation with fluids and a combination of VPA and BA.

Preparation and anesthesia

Preparation

One hour before the start of the experiment, they were premedicated/sedated with an intramuscular injection of midazolam (0.6 mg/kg), ketamine (20 mg/kg), and atropine sulfate (0.05 mg/kg). The animals were placed on the surgical table in a supine position and secured with elastic straps on all four limbs. A peripheral vein on the outer surface of the ear was catheterized with a 20-gauge venous catheter for the administration of induction anesthesia drugs.

Anesthesia

General anesthesia was induced with a bolus intravenous injection of propofol 1-3 mg/kg, fentanyl 0.0025 mg/kg, and cisatracurium 0.5 mg/kg.

Endotracheal intubation was performed using a No. 6.0 endotracheal tube. Subsequently, controlled volume mechanical ventilation was provided via a respirator, with initial ventilation settings of 40% fraction of inspired oxygen (FiO2) in an air mixture, tidal volume (TV) of 10-15 ml/kg, and a respiratory rate (RR) of 18/min. These settings were adjusted to maintain normal blood gas levels and end-tidal carbon dioxide (EtCO2) at 30-40 mmHg. A Levin tube was placed.

Anesthesia was maintained with an intravenous infusion of propofol at a dosage of 10-20 mg/kg/h, fentanyl at 6-10 µg/kg/h for analgesia, and cisatracurium at 1-2 mg/kg/h for muscle relaxation.

Hemodynamic monitoring

A pulse oximeter was placed to monitor oxygen saturation and heart rate, and a digital thermometer was placed in the rectum to monitor body temperature. A vertical incision was made on the right side of the neck, exposing the right external jugular vein, and a single-lumen catheter was placed for blood sampling and fluid/drug administration. The right internal jugular vein was also exposed and cannulated proximally for monitoring the central venous pressure. The right common carotid artery was exposed and catheterized for invasive blood pressure measurement and blood gas analysis. Hourly urine output was measured through a catheter in the cystostomy, performed after the laparotomy.

Surgical protocol

A laparotomy was performed with a midline incision, followed by a cystostomy where a Folley No. 16 catheter was placed. Subsequently, a resection of the left liver lobe weighing approximately 100 grams was performed. This was followed by the controlled loss, using atraumatic vascular clamps, of a specific volume of blood from the traumatic surface of the liver until the mean arterial pressure (MAP) dropped to between 30 and 40 mmHg, which corresponds to approximately 50% total blood volume loss, or 33 ml per kg. The procedure lasted 10 minutes. The controlled hemorrhage from the liver is achieved through intermittent release of hepatic tissue compression. Blood was collected in a container using a suction cannula. The shock was maintained for 50 minutes, and the cut liver surface was sutured with hemostatic sutures. *(During hemorrhage, only anesthesia solutions were administered to the animals.)*

Resuscitation protocol

After 50 minutes of shock, resuscitation began with the administration of warm solutions (~37°C) of crystalloid: sodium chloride 0.9% (NaCl 0.9%) and colloid solutions: 6% hydroxyethyl starch 130/0.4 in 0.9% sodium chloride solution (Voluven, Fresenius Kabi Hellas A.E., Greece) to all animals over a period of 60 minutes, with the goal of returning MAP values to pre-hemorrhage levels +/- 10%. Initially, the administration of fluids was at the maximum rate and was done through the central venous catheter until the desired MAP levels were achieved. The resuscitation fluid administration was initially standardized per kilogram of body weight, and then, if the goal of restoring the MAP to the target range was not met, additional fluids were administered as needed. During resuscitation, the volume of fluids (crystalloids, colloids, and anesthesia solutions) administered was recorded in detail. After the resuscitation phase, the experimental animals received a maintenance solution of D5W 1000 ml + 30 cc NaCl 15% at a rate of approximately 250 ml/h.

Pharmaceutical agents: administration protocol

Group B received BA (bucillamine, carbosynth). Dosage administration: 15 mg/kg/h as a continuous IV infusion throughout the resuscitation period (total of 60 minutes) [11]. Group C received a VPA (Hexaquin, Demo). Dosage administration: 300 mg/kg/h as a continuous IV infusion throughout the resuscitation period (total of 60 minutes) [12]. Group D received both BA and VPA of the dosage mentioned above.

Measurements

The monitoring included recording the following parameters: end-tidal carbon dioxide (EtCO2), oxygen saturation (SpO2), arterial pressure (AP), central venous pressure (CVP), fraction of inspired oxygen (FiO2), tidal volume (TV), respiratory rate (RR), inspiratory-to-expiratory ratio (I/E), heart rate (HR), and rectal temperature. Hourly urine output was also monitored.

To assess liver function/damage, measurements were taken for aspartate aminotransferase (AST), alanine aminotransferase (ALT), and albumin. Interleukin IL-6 was also measured.

**Figure 1:**
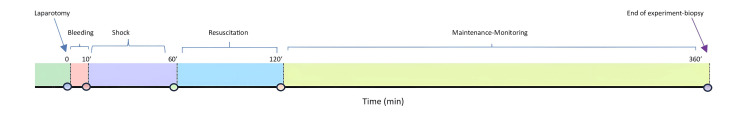
Experimental timeline

Blood samples were taken from the external jugular catheter at the following times: 0 minutes (start of the experiment); 60 minutes (end of HS); 120 minutes (after resuscitation from shock); and 360 minutes (end of the experiment) (Figure 1).

Specifically, blood was collected in vacuum test tubes, centrifuged at 4500 rpm for five minutes, and the serum was stored at -80°C. At the same time, arterial blood was taken from the catheterized carotid artery to measure blood gases and lactic acid.

At the end of the experiment, biopsies were taken from the right lobe of the liver. The liver tissues were divided into two parts. One part was immediately frozen in liquid nitrogen and stored at -80°C, while the other was fixed in formaldehyde solution and then stabilized in paraffin.

Evaluation of oxidative stress and biochemical biomarkers: protein carbonyls (PC) were measured using the method of Levine et al. [13]. The thiobarbituric acid reactive substances (TBARS) assay was used to estimate liver tissue lipid peroxides.

Euthanasia

In accordance with international regulations, governing experimental research on laboratory animals, euthanasia was performed via intravenous administration of 1-2 grams of pentobarbital. Afterward, an autopsy was conducted to check for any surgical complications that could have influenced the measurement results.

Statistical analysis

Data were presented as mean ± standard deviation (SD) for continuous variables, median and interquartile range (IQR: min-max) for ordinal variables, and frequencies (%) for categorical variables. The Shapiro-Wilks test was employed to assess the normality of the distributions. We utilized a two-way mixed ANOVA model using the 'intervention' as the between-groups factor and 'time' as the within-groups factor. The Bonferroni correction was applied to all pairwise comparisons, both between and within groups, to control for multiple testing errors. A sensitivity analysis for the baseline balance between groups was performed by calculating the percentage changes from baseline to each subsequent time point. These changes were analyzed using a one-way ANOVA model. Pairwise comparisons were conducted using the Bonferroni test, and for data that did not meet the assumptions of normality, the Kruskal-Wallis and Mann-Whitney tests were used. For ordinal and categorical data, group comparisons were performed using the Kruskal-Wallis test with Dunn's test for pairwise comparisons, adjusted by the Benjamini-Hochberg False Discovery Rate (FDR) method and the chi-square test, respectively. All tests are two-sided; statistical significance was set at p < 0.05. Analyses were conducted using IBM SPSS Statistics for Windows, Version 21 (Released 2012; IBM Corp., Armonk, New York, United States)

## Results

Hemodynamic and descriptive variables

There was no statistically significant difference in animal weight between the groups. Additionally, blood loss and the mass of the excised liver were similar across all groups (Table 1).

**Table 1:** Descriptive variables between groups Analysis was performed using the one-way ANOVA model.

	Weight (kg)	Excised liver mass (gr)	Blood loss (ml)
Group A	32.83±3.74	102.86±6.99	764.29±224.93
Group B	31.40±1.29	95.00±8.02	718.75±181.14
Group C	33.14±1.99	115.00±10.35	675.00±212.13
Group D	31.19±2.14	98.57±31.05	582.86±110.56
p-value	0.306	0.119	0.332

**Figure 2:**
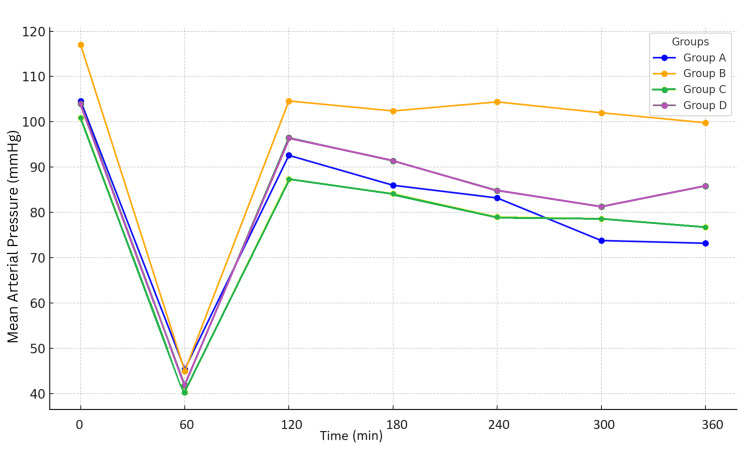
Variation of the mean arterial pressure throughout the experiment for Groups A, B, C, and D (data are expressed as mean ±SD) Although all groups exhibit a similar pattern of an initial sharp decrease in mean arterial pressure (MAP) followed by a recovery phase, differences emerge during the stabilization period. Group B demonstrates the highest sustained MAP, suggesting a more effective intervention, while Group A shows the least favorable outcome. This pattern highlights the initial uniform response to shock across all groups, with subsequent divergence likely reflecting the varying efficacy of the interventions applied.

The mean arterial pressure variations are depicted in Figure 2. 

Fluid requirements for resuscitation

As we described earlier, resuscitation was carried out using crystalloid fluids, specifically isotonic sodium chloride solution (NaCl 0.9%) and colloid fluids, HES 6% (Voluven). The amount of dextrose 5% with NaCl 0.45% was nearly consistent across all four groups and was administered at a steady rate of approximately 250 mL/h following the conclusion of resuscitation.

**Figure 3:**
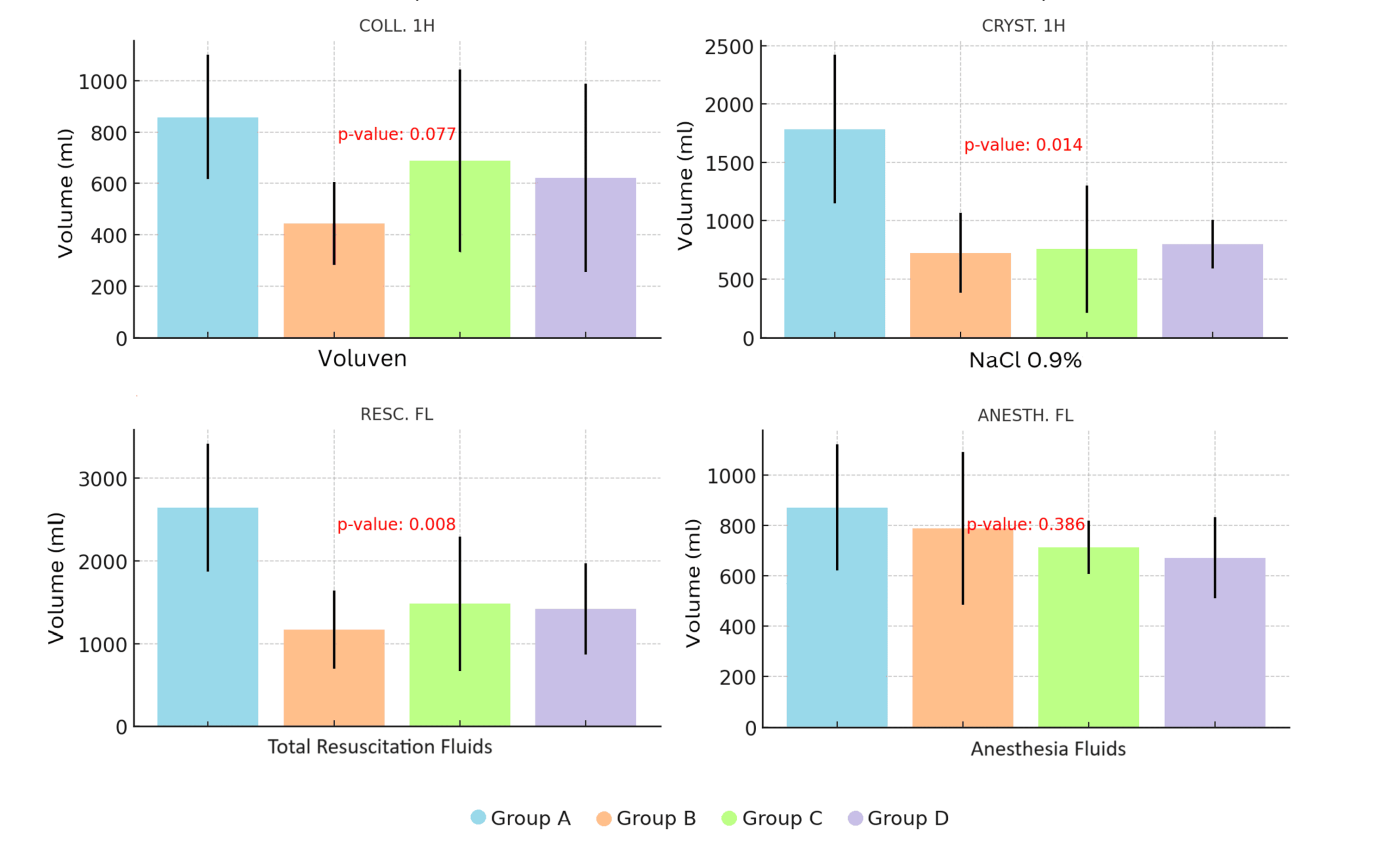
The volume of fluid required across different groups COLL 1H: the volume of HES 6% administered during 60 min of resuscitation, CRYST 1H: the volume of NaCl 0.9% administered during 60 min of resuscitation, RESC FL: total amount of fluids administered during 60 min of resuscitation, ANESTH FL: fluids containing anesthetic drugs during 360 min. Analysis was performed using the one-way ANOVA model.

Our findings indicate that animals treated with BA, VPA, and their combination (Groups B, C, and D) required significantly lower volumes of crystalloid fluids (NaCl 0.9%) during the resuscitation period to restore hemodynamic stability compared to the control Group A (p=0.014). Although the need for colloid solution (Voluven) was also reduced in these groups, the difference did not reach statistical significance (p=0.077). Overall, the total amount of fluids required for resuscitation (RESC. FL) was significantly lower (p=0.008) for the animals of the groups that received treatment. (The volume of fluids containing anesthetic drugs administered throughout the experimental procedure was comparable across all groups, Figure 3).

Liver biochemistry and lactic acid

Alanine aminotransferase (ALT) levels were significantly lower at the end of the experiment (360 min) compared to baseline (0 min) across all groups. However, the percentage decrease from 0 min to 360 min among the different groups was not statistically significant (p=0.325).

Aspartate aminotransferase (AST) levels increased during the experiment, with a statistically significant rise observed only in Group C (p=0.015). The percentage change from 0 min to 360 min across the groups was not statistically significant (p=0.352).

Lactic acid (LAC) levels were significantly elevated across all groups at the end of the experiment. The percentage change from 0 min to 360 min across the groups was statistically significant (p=0.016). From the multiple comparisons between all groups, we noted that Group B showed a significantly lower increase (56.42%) in LAC levels compared to Groups A: 215.61%, C: 298.70%, and D: 267.50% at the end of the experiment (Table 2).

**Table 2:** Lactic acid levels and hepatic transaminases, mean values ±SD (at the beginning and the end of the experiment, 0 and 360 min, respectively) 0 min: start of the experiment; 360 min: end of the experiment ALT: alanine aminotransferase (U/L); AST: aspartate aminotransferase (U/L); LAC: lactic acid (mmol/L) *p-value: indicates the p-value comparing the difference of each parameter from time 0 to 360 minutes within each group (intragroup variance). p.c. -360 min (%): Represents the percentage change of the measured parameter from time 0 to 360 minutes. **p-value: represents the p-value comparing the percentage change between all groups. A two-way ANOVA model was used to assess within-group differences over time. One-way ANOVA, including the Bonferroni test for post-hoc analysis, was used to compare percentage changes between groups.

Parameter	Group	0 min	360min	*p-value	p.c. 0-360 (%)	**p-value
ALT (U/L)	A	44.30 ± 8.03	26.13 ± 7.37	0.001	-41.34 ± 12.00	
	B	32.91 ± 8.53	22.96 ± 5.08	0.025	-28.55 ± 14.10	0.325
	C	40.39 ± 10.79	24.66 ± 10.25	0.001	-40.70 ± 16.93	
	D	31.51 ± 10.67	18.50 ± 11.36	0.046	-42.95 ± 22.53	
AST (U/L)	A	24.49 ± 4.64	94.2 ± 36.35	0.29	284.69 ± 120.50	
	B	55.07 ± 36.93	110.57 ± 42.17	0.33	100.78 ± 75.25	0.352
	C	31.79 ± 9.82	93.68 ± 70.62	0.015	194.69 ± 96.13	
	D	33.41 ± 15.17	76.8 ± 42.54	0.19	129.85 ± 85.45	
LAC (mmol/L)	A	1.10 ± 0.45	3.56 ± 2.02	0.001	215.61 ± 127.86	
	B	1.09 ± 1.11	1.30 ± 0.83	0.005	56.42 ± 100.51	0.016
	C	1.16 ± 0.74	3.66 ± 1.22	<0.001	298.70 ± 222.75	
	D	0.89 ± 0.39	2.83 ± 0.79	<0.001	267.50 ± 160.57	

Oxidative stress markers

Serum lipid peroxides were estimated using the thiobarbituric acid reactive substances (TBARS) assay, and serum protein carbonyls (PCs) were determined by the method of Levine et al. [13].

**Figure 4:**
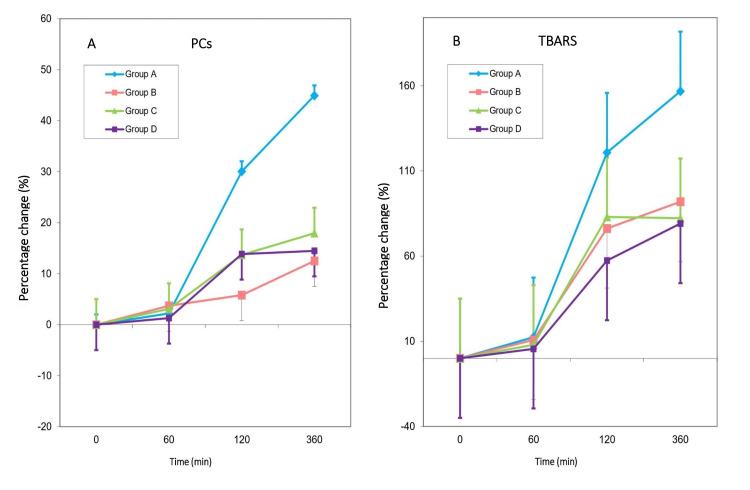
(A) Percentage change in serum PCs from baseline over time; (B) Percentage change in serum TBARS from baseline over time A. No statistically significant differences were among the four groups for changes from time 0 to 60 minutes (p=0.290). However, significant differences were observed at 120 minutes and 360 minutes (both p<0.05). Group A differed from Groups B, C, and D at both 120 and 360 minutes, while no differences were found among Groups B, C, and D. B. No statistically significant differences among the four groups for percentage change in TBARS from time 0 to 60 minutes (p=0.594). However, significant differences were observed at 120 minutes (p=0.007) and 360 minutes (p=0.005). At 120 minutes, Group A differed from Groups B (p=0.045), C (p=0.055), and D (p<0.005), with no differences between Groups B, C, and D. Similarly, at 360 minutes, Group A differed from Groups B (p=0.054), C (p=0.011), and D (p=0.011), while no differences were found among Groups B, C, and D. Analysis was conducted using the one-way ANOVA model with the Bonferroni correction for the pairwise comparisons.

We examined the effect of the therapeutic interventions by comparing the percentage change of the serum PCs and TBARS from time 0 at each time point of the study among the groups (Figure 4).

Histological examination

Liver specimens embedded in paraffin wax were sectioned into 4μm slices and stained with hematoxylin and eosin (H&E). The first and third authors of this paper blindly and independently assessed the specimens, using a Zeiss microscope, to identify signs of hepatocellular damage, specifically assessing indices such as inflammation, steatosis, necrosis, and more (Tables 3, 4, 5). *(Inter-observer variability was verified using the linear weighted-kappa value and evaluated according to Landis and Koch guidelines.)*

**Table 3:** Histologic scoring system

Score	0	1	2	3
Periportal inflammation	None	Mild	Moderate	Severe
Mid-lobular inflammation	None	Mild	Moderate	Severe
Periportal necrosis	None	<30%	30-60%	>60%
Mid-lobular necrosis	None	<30%	30-60%	>60%
Centrilobular necrosis	None	<30%	30-60%	>60%

**Table 4:** Histological results of categorical data Categorical data are expressed as frequencies (percent). Analysis between groups was performed using the chi-square test.

Parameter	Group A (n=6)	Group B (n=7)	Group C (n=6)	Group D (n=8)	p-value
Disruption of hepatic lobular architecture (%)	6(100.0%)	7(100.0%)	6(100.0%)	8(100.0%)	1.000
Apoptotic bodies (%)	3(50.0%)	0(0.0%)	2(33.3%)	2(25.0%)	0.218
Steatosis (%)	1(16.7%)	0(0.0%)	4(66.7%)	3(37.5%)	0.055

**Table 5:** Histological results of ordinal data Ordinal data are expressed as median and IQR (lower value-upper value), *p-values < 0.05. Analysis was performed using the Kruskal-Wallis with Dunn test adjusted by the Benjamini-Hochberg False Discovery Rate (FDR) method.

Parameter	Group A	Group B	Group C	Group D	p-value
Congestion	0.5 (0–2)	0.0 (0–1)	0.0 (0–1)	0.0 (0–2)	0.812
Expansion of Disse's space	0.5 (0–2)	0.0 (0–1)	0.0 (0–1)	0.0 (0–2)	0.812
Sinusoidal dilatation	1.0 (1–1)	2.0 (0–2)	2.0 (1–2)	1.5 (1–2)	0.062
Central lobe vein distension	1.0 (0–1)	1.0 (0–2)	2.0 (1–2)	1.0 (0–2)	0.087
Widening of portal space	0.5 (0–2)	0.0 (0–1)	1.0 (0–2)	1.0 (1–2)	0.066
Periportal inflammation	1.5 (0–2)	1.0 (0–2)	1.5 (0–2)	1.0 (0–2)	0.731
Mid-lobular inflammation	2.0 (1–3)	1.0 (0–1)	0.5 (0–1)	1.0 (0–1)	0.012*
Periportal necrosis	1.0 (1–2)	0.0 (0–1)	0.0 (0–1)	0.0 (0–1)	0.010*
Centrilobular necrosis	1.5 (1–2)	0.0 (0–1)	0.5 (0–1)	0.0 (0–1)	0.007*
Mid-lobular necrosis	0.5 (0–2)	0.0 (0–1)	0.0 (0–1)	0.0 (0–3)	0.574

Observations: significant findings

In what follows, we present the most significant findings of the histological evaluation. Certain parameters, such as apoptotic bodies, congestion, Disse’s space expansion, sinusoidal dilatation, central lobe vein distension, widening of portal spaces, and periportal inflammation, did not show statistically significant differences. However, there was a borderline statistically significant difference regarding the presence of steatosis between the compared groups (p=0.055), Table 4. From the pairwise comparisons, Group C had a higher percentage of steatosis compared to Group B (66.7% vs. 0%, p=0.021). We did not find statistically significant differences between all other groups.

**Figure 5:**
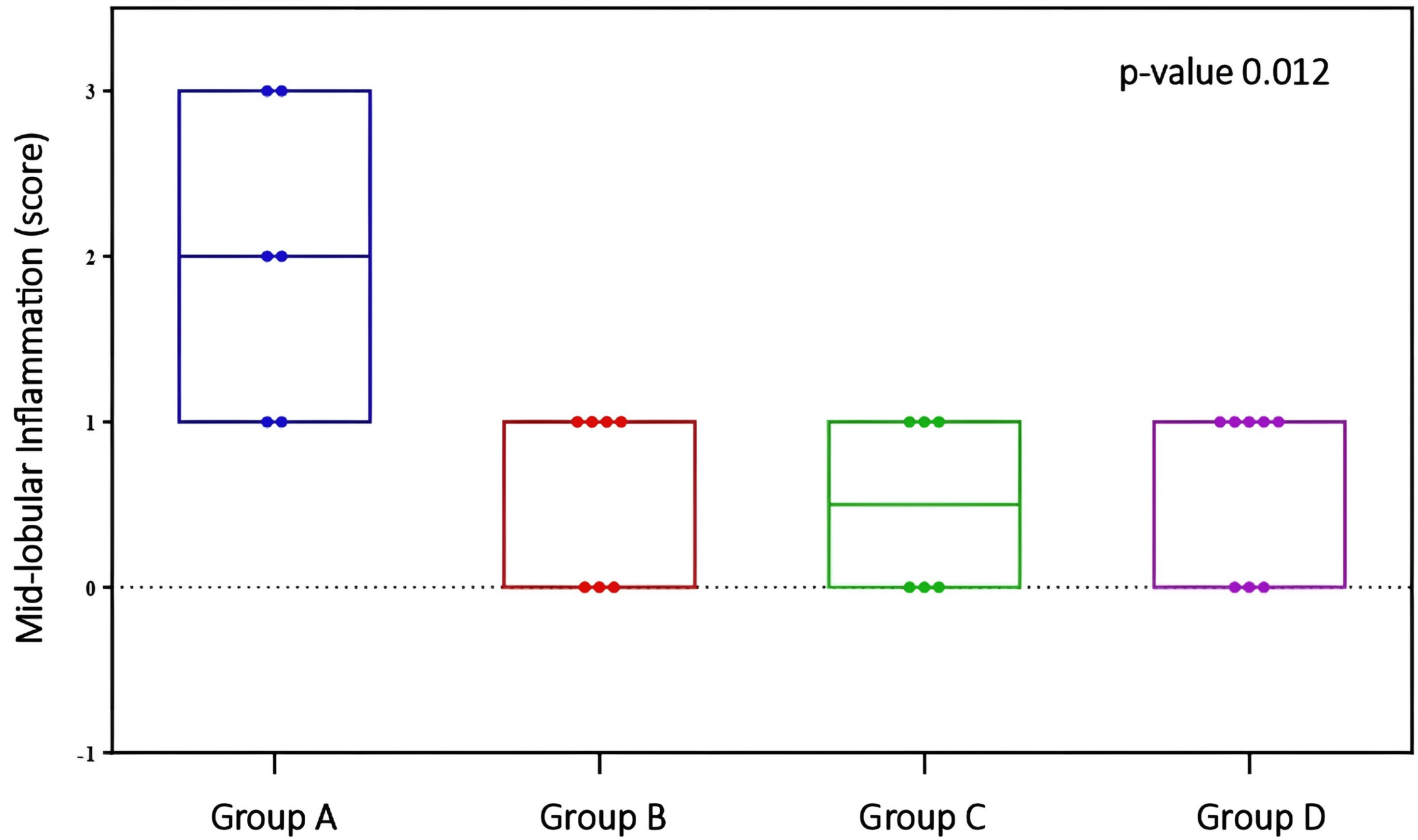
Comparison of mid-lobular inflammation between groups Mid-lobular Inflammation: p-index=0.012. Pairwise comparison between Group A and Groups B (p-index=0.038), C (p-index=0.028), and D (p-index=0.048). Analysis was performed using the Kruskal-Wallis with Dunn test adjusted by the Benjamini-Hochberg False Discovery Rate (FDR) method.

There was a statistically significant difference between the groups regarding the ‘mid-lobular inflammation’ parameter (p=0.012). Pairwise comparisons revealed differences between Group A and Groups B (p=0.038), C (p=0.028), and D (p=0.048), respectively (Table 5 and Figure 5).

**Figure 6:**
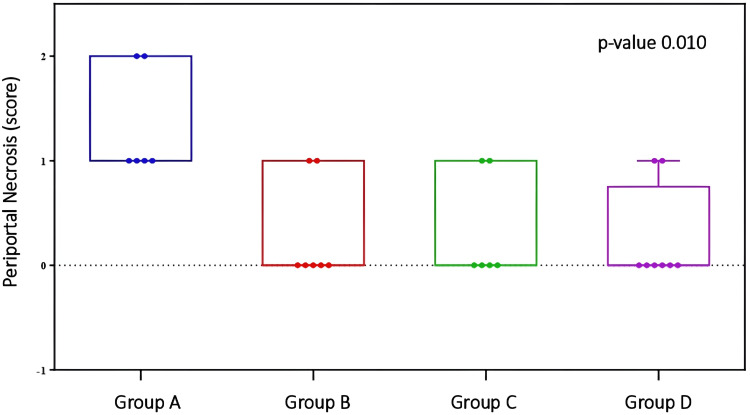
Comparison of periportal necrosis between groups Periportal necrosis: p-index=0.010. Pairwise comparisons between Group A and Groups B (p-index=0.030), C (p-index=0.044), and D (p-index=0.016). Analysis was performed using the Kruskal-Wallis with Dunn test adjusted by the Benjamini-Hochberg False Discovery Rate (FDR) method.

There was a statistically significant difference between the groups regarding the ‘periportal necrosis’ parameter (p=0.010). Pairwise comparisons revealed differences between Group A and Groups B (p=0.030), C (p=0.044), and D (p=0.016), respectively (Table 5 and Figure 6).

**Figure 7:**
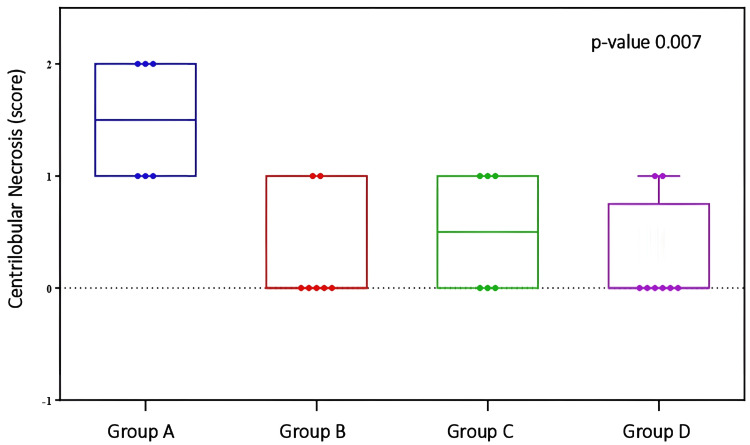
Comparison of Centrilobular necrosis necrosis between groups Centrilobular necrosis: p-index=0.007. Pairwise comparison between Group A and Groups B (p-index=0.003), C (p-index=0.025), and D (p-index=0.009). Analysis was performed using the Kruskal-Wallis with Dunn test adjusted by the Benjamini-Hochberg False Discovery Rate (FDR) method.

There was also a statistically significant difference between the groups regarding the ‘centrilobular necrosis’ parameter (p=0.007). Pairwise comparisons revealed differences between Group A and Groups B (p=0.003), C (p=0.025), and D (p=0.009), respectively (Table 5 and Figure 7).

Overall, Group A exhibited significantly more severe histological damage in the liver compared to the other groups. The most pronounced findings were widespread inflammation within the liver lobules, characterized by marked infiltration of inflammatory cells, periportal necrosis, as well as necrosis in the centrilobular region of the liver, which is particularly vulnerable to ischemic damage due to its location, furthest from the blood supply provided by the hepatic artery and portal vein.

Table 4 and Table 5 show the histological features of the liver tissues that were examined. (Due to technical issues during the H&E staining process, three animals were excluded from the histological analysis, one from Group B and two from Group C.)

Ordinal data are presented in Table 5 as the median and interquartile range (IQR).

**Figure 8:**
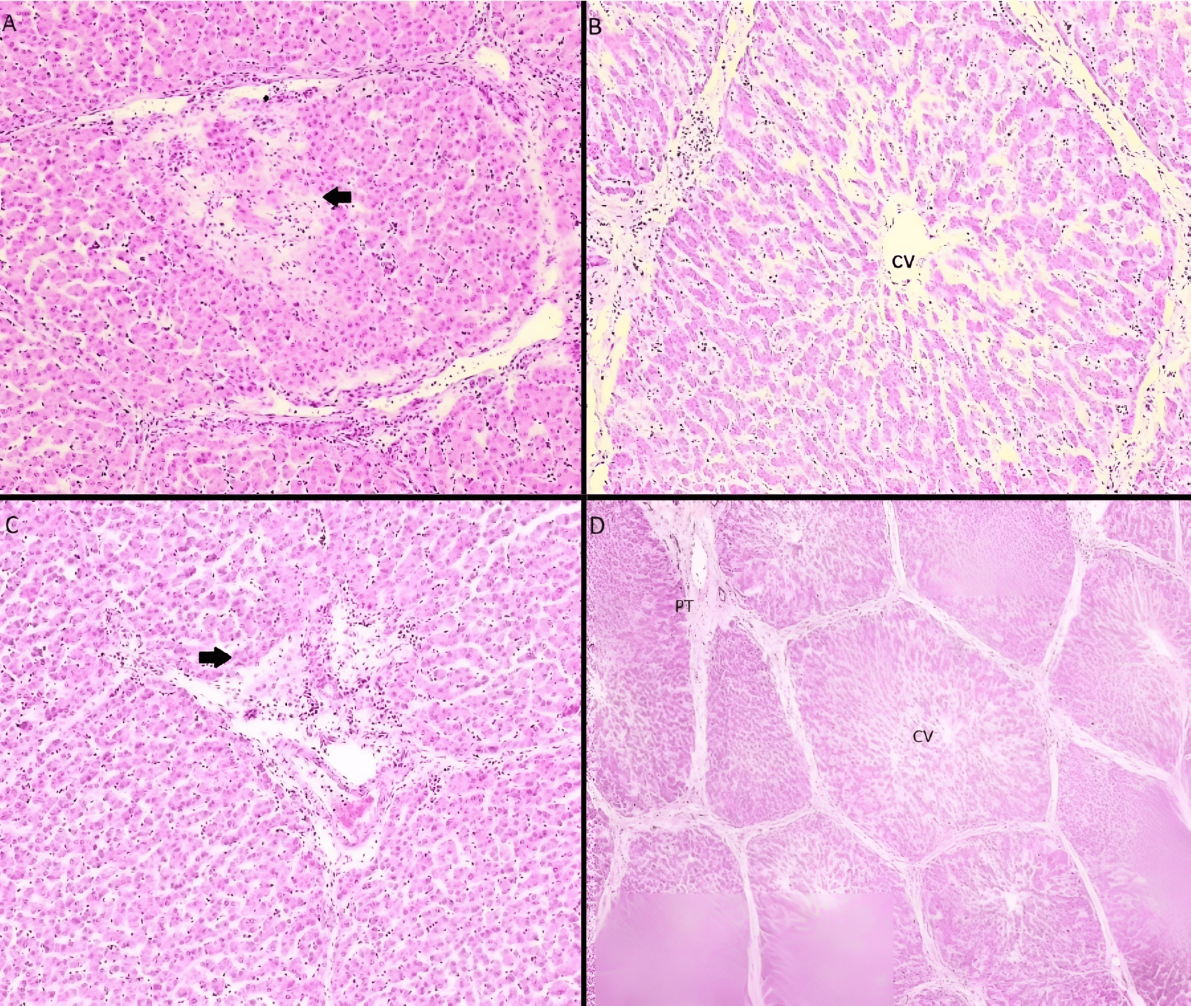
Microphotographs of liver tissues A. Centrilobular necrosis and inflammation. The necrotic hepatocytes are seen as slightly more eosinophilic (red) and discohesive. The necrotic hepatocytes have barely discernible nuclei. Note the paler staining of this area compared to the surrounding tissue (Group A, original magnification x40). B. We notice a slight distention of the central vein (CV) without signs of centrilobular necrosis or inflammation (Group B, original magnification x100). C. Periportal concentration of inflammatory cells. These cells are smaller, round, and darker-stained (deep purple), clustering within the portal region. Early necrotic changes and degeneration of hepatocytes are also noted (pale region-arrow) (Group C, original magnification x100). D. The liver lobules are clearly delineated by fibrous septa, which is a normal anatomical feature in swine liver. The central vein (CV) of each lobule is visible, surrounded by hepatocytes arranged radially. There are no signs of necrosis or inflammation; slight sinusoidal dilatation is present (Group D, original magnification x10).

The microphotographs below, demonstrate the most characteristic lesions in the hepatic tissue that we discovered and evaluated in the study (Figure 8).

## Discussion

The future of pharmaceutical treatment for HS in major hepatic surgery or trauma lies in the development of more targeted therapies with fewer side effects. Research into novel hemostatic agents, personalized medicine approaches, and enhanced monitoring technologies holds promise for improving outcomes in this challenging clinical setting [14]. Recent advancements highlight the potential of new hemostatic agents and advanced resuscitation strategies to manage HS more effectively [15].

IRI, a common occurrence in HS, leads to the production of ROS, causing oxidative stress, which induces cellular damage through mechanisms such as lipid peroxidation, protein oxidation, and DNA damage, contributing to apoptosis and necrosis [16,17]. The administration of large volumes of crystalloids and colloids during resuscitation can exacerbate these effects by diluting clotting factors and causing further tissue edema and inflammation [18,19].

In vivo experiments, swine models were thoroughly performed concerning hepatic IRI. Arkadopoulos et al. showed that the use of deferoxamine (DFO) can ameliorate IRI during major hepatectomies performed under vascular exclusion of the liver [20]. In addition, Paradellis et al. also reported that the administration of DFO had an early protective effect against liver IRI when performing major hepatectomies [21]. Furthermore, Orfanos et al., by administrating N-acetylcysteine and DFO in a swine model of liver hemorrhage, concluded that it may reduce the fluid amounts needed for resuscitation, and this antioxidant combination restores the energy-dependent apoptosis and proliferation of the hepatocytes [22]. Mylonas et al. investigated the differing biological responses to HS from hepatic versus peripheral hemorrhage, finding that hepatic hemorrhage leads to worse hepatocyte outcomes, including higher resuscitation fluid needs, reduced cell proliferation, and increased inflammation with potential clinical implications for liver surgery and trauma [23].

Our experimental study, also performed in a swine model as described above, aimed to evaluate the efficacy of combining BA and VPA in mitigating the adverse effects of HS following partial hepatectomy.

BA is a chemical compound structurally analogous to cysteine, containing two thiol groups that provide it with a stronger antioxidant effect compared to other cysteine derivatives, such as NAC. Clinically, it is administered orally to treat rheumatoid arthritis [24]. As an antioxidant, BA maintains high levels of glutathione (GSH), a crucial intracellular antioxidant, and exhibits immunomodulatory effects by suppressing the production of the pro-inflammatory cytokine IL-6. Additionally, its chelating properties help delay iron-induced free radical production [25].

Known for its antiepileptic properties, VPA has shown promise in improving survival rates in experimental models of HS. It inhibits histone deacetylases (HDACs), leading to the hyperacetylation of histones and the transcription of genes associated with cell survival and apoptosis inhibition. This epigenetic modulation helps mitigate the effects of IRI by reducing inflammation and oxidative stress. VPA, known as an antiepileptic drug, has been used in several experimental models of hemorrhage, showing improved survival after HS [26]. The exact mechanism of its action is not fully understood, but it is known that at high doses, it inhibits the activity of histone deacetylases (HDAC) 1, 2, 3, and 8, and induces the acetylation of histones and other proteins. Histone acetylation is a post-transcriptional modification that causes changes in chromatin structure, inducing transcription and gene expression [27]. Experimental studies have shown that hemorrhage and its resuscitation are processes biologically associated with an imbalance between the actions of two enzymes responsible for nuclear histone acetylation: histone acetyltransferases (HATs) and histone deacetylases (HDACs). Various resuscitation strategies affect the degree of histone acetylation in organs like the heart and liver. Moreover, the addition of deacetylase inhibitors to resuscitation solutions in conditions of HS causes hyperacetylation of histones and induces the transcription of genes related to apoptosis (Bcl-2, surviving, c-myc, JNK pathway) [28,29]. The use of VPA as an additional treatment during resuscitation in a swine model of hemorrhage and IRI showed significant advantages by reducing the need for crystalloids while enhancing physiological and hemodynamic outcomes [30].

The present study showed that the use, as well as the combination of BA and VPA, significantly reduced markers of oxidative stress and inflammation in the liver, suggesting a protective effect against hemorrhagic shock-induced damage. The therapy also demonstrated the potential to improve hemodynamic stability and reduce the volume of fluids required for resuscitation, thereby minimizing the risk of fluid overload and secondary complications.

Our results highlight the potential of targeted pharmaceutical interventions in managing HS during hepatic trauma or surgery. In particular, the use of antioxidants and HDAC inhibitors offers a promising strategy to reduce oxidative stress and possibly improve clinical outcomes. Although we had anticipated more promising results from the combination of BA and VPA administered to Group D, these expectations were ultimately not met. Therefore, we believe that further research is needed to optimize dosing regimens and explore the long-term effects of these treatments in experimental settings.

Finally, a key limitation of the study was the short duration of post-resuscitation follow-up. Cellular events such as apoptosis and cell proliferation often unfold over hours to days; thus, extending the observation period to 12 or even 48 hours could provide a more comprehensive understanding of the liver's regenerative and apoptotic responses following hemorrhagic shock and resuscitation. Such an extended timeframe would yield more reliable conclusions about the long-term effects of the interventions on hepatic cellular dynamics.

## Conclusions

In conclusion, the pharmaceutical treatment of hemorrhagic shock in hepatic surgery and/or trauma is a complex and evolving field. The combination of vasopressors, hemostatic agents, blood products, and advanced monitoring techniques has significantly improved critically ill patient outcomes. Moreover, recent studies suggest that antioxidant drugs may play a potential role in mitigating oxidative stress and reducing tissue damage. By neutralizing free radicals and reducing inflammatory responses, antioxidants can potentially help preserve cellular function, particularly in the liver, which is highly susceptible to ischemia-reperfusion injury. Continued research, innovation, and gaining a deeper insight into the pathophysiology of hemorrhagic shock are essential to further enhance the safety and efficacy of these treatments.
